# Ocular biomarkers: useful incidental findings by deep learning algorithms in fundus photographs

**DOI:** 10.1038/s41433-024-03085-2

**Published:** 2024-05-11

**Authors:** Eve Martin, Angus G. Cook, Shaun M. Frost, Angus W. Turner, Fred K. Chen, Ian L. McAllister, Janis M. Nolde, Markus P. Schlaich

**Affiliations:** 1 Commonwealth Scientific and Industrial Research Organisation (CSIRO), Kensington, WA Australia; 2 School of Population and Global Health, The University of Western Australia, Crawley, Australia; 3 Dobney Hypertension Centre - Royal Perth Hospital Unit, Medical School, The University of Western Australia, Perth, Australia; 4 Australian e-Health Research Centre, Floreat, WA Australia; 5 Lions Eye Institute, Nedlands, WA Australia; 6 Centre for Ophthalmology and Visual Science, The University of Western Australia, Perth, Australia; 7 Centre for Eye Research Australia, The Royal Victorian Eye and Ear Hospital, East Melbourne, VIC Australia; 8 Ophthalmology, Department of Surgery, The University of Melbourne, East Melbourne, VIC Australia; 9 Ophthalmology Department, Royal Perth Hospital, Perth, Australia; 10 Departments of Cardiology and Nephrology, Royal Perth Hospital, Perth, Australia

**Keywords:** Prognostic markers, Public health, Retinal diseases, Prognostic markers

## Abstract

**Background/Objectives:**

Artificial intelligence can assist with ocular image analysis for screening and diagnosis, but it is not yet capable of autonomous full-spectrum screening. Hypothetically, false-positive results may have unrealized screening potential arising from signals persisting despite training and/or ambiguous signals such as from biomarker overlap or high comorbidity. The study aimed to explore the potential to detect clinically useful incidental ocular biomarkers by screening fundus photographs of hypertensive adults using diabetic deep learning algorithms.

**Subjects/Methods:**

Patients referred for treatment-resistant hypertension were imaged at a hospital unit in Perth, Australia, between 2016 and 2022. The same 45° colour fundus photograph selected for each of the 433 participants imaged was processed by three deep learning algorithms. Two expert retinal specialists graded all false-positive results for diabetic retinopathy in non-diabetic participants.

**Results:**

Of the 29 non-diabetic participants misclassified as positive for diabetic retinopathy, 28 (97%) had clinically useful retinal biomarkers. The models designed to screen for fewer diseases captured more incidental disease. All three algorithms showed a positive correlation between severity of hypertensive retinopathy and misclassified diabetic retinopathy.

**Conclusions:**

The results suggest that diabetic deep learning models may be responsive to hypertensive and other clinically useful retinal biomarkers within an at-risk, hypertensive cohort. Observing that models trained for fewer diseases captured more incidental pathology increases confidence in signalling hypotheses aligned with using self-supervised learning to develop autonomous comprehensive screening. Meanwhile, non-referable and false-positive outputs of other deep learning screening models could be explored for immediate clinical use in other populations.

## Introduction

### Screening with deep learning

Since 2018 when the first autonomous machine learning model was approved to detect diabetic retinopathy in fundus photographs [1], deep learning algorithms have now expanded to assist diagnosis of macular degeneration [2] and glaucoma [3]. Regulatory approval is also pending for myopic retinopathy [4] and cardiovascular disease [5]. Deep learning algorithms can outperform human accuracy for the three targeted diseases: diabetic retinopathy/diabetic macular oedema [6, 7], macular degeneration [8], and glaucoma [9]. The technology is more accessible [10–13], 200 times faster [14, 15], and capable of sub-clinical detection [16–19], but the diseases screened are few and human experts are still required for complete retinal assessment.

The only clinically available systemic disease targeted by deep learning in fundus photographs remains diabetes mellitus despite expanding algorithm development to estimate age [20], refractive error [21], smoking status [22], body composition [23], renal function [23], glycated haemoglobin levels [24], anaemia [25], schizophrenia [26], neurodegenerative diseases [27, 28] as well as cardiovascular [22, 29, 30] and cerebrovascular health [31, 32]. Multi-target algorithms [33–35] and multimodality using scanning laser imaging and OCT technology [36] are also rapidly advancing, but realising autonomous comprehensive retinal screening on this trajectory is unlikely as the required training datasets for rare and novel diseases are lacking, and target-specific algorithms are not designed to detect clinically important incidental diseases noticed by human experts.

### Applications in hypertensive and diabetic retinopathies

Retinal photography, the most effective screening strategy for diabetic retinopathy [37], shows the extent of progressive retinal vessel compromise and estimates the health of the other target organs; the heart, brain, and kidneys [38]. Similarly, the extent of hypertensive retinal vascular remodelling estimates the health of the other target organs [39]. Unlike the management of diabetes mellitus, where glycated haemoglobin provides time-averaged blood glucose estimates, hypertension has no such time-averaged metric to compensate for the volatility of blood pressure. Thus, hypertensive ocular biomarkers are key indicators for non-invasive management and offer preclinical detection, as arteriolar narrowing occurs prior to clinical hypertension [40].

More than twice as prevalent as diabetes mellitus, hypertension, defined as office blood pressure levels above 140 mmHg systolic, 90 mmHg diastolic, or both [41] remains the leading modifiable risk factor for premature death worldwide [42], affecting 20% of the global adult population, with 46% undiagnosed and only 21% receiving effective treatment [43]. Interestingly, clinical standards of care call for ongoing screening and monitoring of diabetic retinas, whereas there are no such recommendations for hypertension beyond retinal screening at diagnosis [44].

Not only is hypertensive retinopathy the most common clinically significant incidental finding in diabetic screening [45], but hypertensive retinal features appear to dominate the pathologies misclassified by deep learning algorithms [45–59]. Despite exploration of vessel calibre changes [60] and small vessel segmentation using optical coherence tomography angiography (OCTA) [61, 62], difficulties remain differentiating some early biomarkers of diabetic and hypertensive retinopathies [63].

Hypothetically, shared retinal biomarkers and/or high rates of hypertension in diabetic training images [64] could trigger false-positive results for deep learning algorithms. Additionally, or alternatively, training deep learning pattern recognition/discrimination for specific disease is not error-free and may result in anomalous data signals triggering misclassification of not only hypertensive retinopathy but also other clinically useful untargeted disease. Deep learning pathways remain unknown, but the hypotheses suggest that the algorithms may be clinically useful beyond their intended targets.

The primary aim of this study was to explore the potential for detecting clinically useful incidental ocular biomarkers using diabetic deep learning algorithms to screen fundus photographs of hypertensive adults.

## Subjects and methods

### Research design

The study had a retrospective, observational design. Approval was obtained from the East Metro Health Service Ethics and Governance Unit to use images collected for *EastMetro HREC RGS1040 - Retinal Imaging in Resistant Hypertension*. The study adhered to the tenets of the Declaration of Helsinki for research involving human subjects, and all participants gave informed consent.

### Participants

Participants were recruited from Dobney Hypertension Centre, a public hospital outpatient clinic in Western Australia specializing in resistant hypertension, defined as uncontrolled high blood pressure despite at least three antihypertensive medications including a diuretic [41]. All participants were confirmed to have hypertension at recruitment but not necessarily with resistant hypertension.

All patients attending Dobney Hypertension Centre between 18 January 2016 and 31 March 2022 were invited to participate in the *Retina Imaging in Resistant Hypertension* study. Recruitment, data collection and processing have been described in detail previously [65, 66]. In brief, patients were referred from primary care for diagnostic workup and clinical management of difficult-to-control hypertension. Patients consented to participate in a systematic prospective analysis to explore the association between blood pressure at presentation and retinal imaging parameters. Baseline clinical data collected from the patients included medical history, medication history, serum pathology, extensive blood pressure testing, and specific assessments of hypertension-mediated organ damage (including retinal imaging).

The participation rate for all new and returning attendees was above 90%. Of the 529 consented participants, 96 were excluded from the study for lack of imaging arising from technical issues or non-attendance.

### Data collection

#### Image acquisition

Clinic staff collected 45° macula-centred, colour fundus photographs without mydriasis using a Canon CR-2 camera (Tokyo, Japan) along with OCT and OCTA imaging by Optovue Avanti XR (Fremont, California, USA). All images and data were de-identified before transfer to the Commonwealth Scientific and Industrial Research Organisation (CSIRO), Australia’s national science agency.

#### Image selection

For each participant, the most recent processable higher-quality image capturing both the optic nerve and macula was selected. Processability was determined by two or more algorithms successfully processing the image. Two of the algorithms output a graded image or deemed the image *ungradable*, while the third algorithm graded all images along with a quality scale and a reliability threshold. Where no image met the processability criteria, the participant was removed from further assessment. Where more than one image met the criteria, the image with the algorithm-determined higher degree of pathology was selected. The same 45° colour fundus photograph selected for each participant was processed by three deep learning algorithms.

#### Algorithms

Algorithm 1, *DR Grader* (Perth, Australia), was approved in 2018 as a Class I medical device to detect only referable diabetic retinopathy, differentiating moderate and severe. Referable diabetic retinopathy is internationally recognised as *more than mild* diabetic retinopathy [67]. Verified on a data set of 193 images by two human experts for referable diabetic retinopathy, the algorithm identified 17 as positive including the two true positive cases, resulting in 100% capture of true positives and a positive predictive value of 12% [46].

Algorithm 2, *RetCAD* (Nijmegen, The Netherlands), was approved in 2020 as a Class IIa medical device for simultaneous detection of diabetic retinopathy and macular degeneration. The algorithm not only quantified all diabetic retinopathy and macular degeneration results, but also graded fundus photograph quality and estimated vertical cup-to-disk ratio. For all images, Algorithm 2 produced a contrast-enhanced image along with separate precise heatmaps of bright and red lesions. In 2022, in a real-world tertiary hospital screening setting, the algorithm processed 7195 images for referable diabetic retinopathy resulting in 90.5% sensitivity and 97.1% specificity [68]. When mild diabetic retinopathy was included, sensitivity rose to 91.7% and specificity dropped to 90.9% [69].

Algorithm 3, *Eyetelligence* (Melbourne, Australia), was approved in 2019 as a Class I medical device for three separate target diseases: diabetic retinopathy [47], macular degeneration [2], and glaucoma [70]. The algorithm categorised diabetic retinopathy into the four internationally recognised levels of diabetic retinopathy; mild, moderate, severe, and proliferative [67, 71], with a sensitivity of 92.5% and a specificity of 98.5% [47]. Algorithm 3 also output a diffuse heatmap for referable diabetic retinopathy results only.

#### Grading of known false-positive results

Within the images identified by one or more of the algorithms as positive for diabetic retinopathy, 29 participants were verified as clinically non-diabetic, an absence of diabetic or pre-diabetic clinical diagnosis, and a confirmed glycated haemoglobin result below 5.7 mmol/L. The 29 misclassified non-diabetic images were graded by two highly qualified retinal specialists with extensive experience in both clinical practice and research at the Lion’s Eye Institute, Perth. Both assessors independently identified the ocular anomalies visible in the sample. Where diagnostic ambiguities arose, the final determination was made by reviewing additional images, the fellow eye, medical history, heatmaps, OCT, and OCTA scans. The types and relative frequencies of ocular anomalies found in the 29 misclassified images were then recorded as the primary outcome for each algorithm.

## Results

### Sample derivation

Of the 433 participants imaged, 27 images (6%) were of insufficient quality for reliable assessment by two or more algorithms. Of the 406 assessable images, 251 (62%) returned negative results for all target anomalies across all three algorithms.

Of the 155 images returning positive results, 56 were positive for macular degeneration or glaucoma and 99 (63.9%) were flagged as positive for diabetic retinopathy. Of the 99 images, 14 (14%) were flagged by all three algorithms, 28 (28%) were flagged by two algorithms, and 57 (58%) were flagged by one algorithm.

Of the 99 participants with positive diabetic retinopathy results, 48 had a diagnosis of diabetes mellitus or had glycated haemoglobin levels in the diabetic range, and 22 were pre-diabetic based on glycated haemoglobin testing. The remaining 29 participants with positive diabetic retinopathy results (29% of the 99 reported as positive for diabetic retinopathy and 7% of the 406 assessable images) had no diabetic or glucose-control medication history and had glycated haemoglobin levels below 5.7 mmol/L.

Based on clinical evidence, these 29 participants were not at risk for diabetic retinopathy and their images were classified as false-positive results. Their ages ranged from 23 to 90 years (16 males; mean age 63 ± 16 years).

### Features of false-positive results

Table 1 shows the frequency and capture rates by algorithm for the features found by retinal experts in the 29 images misclassified as positive for diabetic retinopathy. With few exceptions, the capture rates for each feature were highest for the single-target Algorithm 1, lower for the dual-target Algorithm 2, and lowest for the triple-target Algorithm 3.

**Table 1:** List of Features in 29 Images Misclassified as Diabetic Retinopathy with Capture Rates.

Features listed with capture rates for 29 images misclassified as positive for diabetic retinopathy				
Total number of images with feature	Features	Model 1	Model 2	Model 3
		*n* = 24	*n* = 13	*n* = 8
(% of 29)	(% of Total Number of Images with Feature)			
21 *(72%)*	*All Hypertensive Retinopathy*	18 *(90%)*	12 *(57%)*	7 *(33%)*
20 *(69%)*	*Arteriolar Narrowing*	18 *(90%)*	10 *(50%)*	6 *(30%)*
6 *(21%)*	*Haemorrhage or Microaneurysm*	4 *(67%)*	5 *(83%)*	4 *(67%)*
6 *(21%)*	*Cotton Wool Spots*	4 *(67%)*	4 *(67%)*	2 *(33%)*
6 *(21%)*	*Lipid Exudates (Macular Star)*	6 *(100%)*	6 *(100%)*	5 *(83%)*
6 *(21%)*	*Tessellation (Myopic, Blonde or Tigroid Fundus)*	6 *(100%)*	1 *(17%)*	
5 *(17%)*	*Retinal Vein Occlusion*	4 *(80%)*	5 *(100%)*	3 *(60%)*
4 *(14%)*	*Hypertensive Disc Swelling*	4 *(100%)*	4 *(100%)*	3 *(75%)*
4 *(14%)*	*Macular Degeneration*	4 (*100%)*		1 *(25%)*
4 *(14%)*	*Scattered or Peripheral Drusen*	4 *(100%)*	1 *(25%)*	
2 *(7%)*	*Macular Pigment Epithelium Disruption*	2 *(100%)*		
1 *(3%)*	*Optic Nerve Pallor*	1 *(100%)*	1 *(100%)*	1 *(100%)*
1 *(3%)*	*Suspect Choroiditis/Malattia Leventinese*	1 (*100%)*		
1 *(3%)*	*Choroidal Naevus*	1 *(100%)*		
1 *(3%)*	*Peau d ’Orange with Angioid Streaks (Pseudoxanthoma Elasticum)*		1 *(100%)*	
1 *(3%)*	*Widened Fovea with Unusual Macular Surface (Suspect Epiretinal Membrane)*	1 *(100%)*	1 *(100%)*	1 (*100%)*
1 *(3%)*	*Cuticular Drusen (associated with Membranoproliferative Glomerulonephritis)*	1 *(100%)*		
1 *(3%)*	*Cataract*	1 (*100%)*		

In column 1, the percentages in italics indicate the total number of images with the feature in column 2 as a percentage of the total 29 images. In columns 3–5, the percentages in italics indicate the number of images with the feature listed in column 2 detected by each model as a percentage of the total number with the feature in column 1.

Supplementary Table 1 lists the features observed by human experts for each of the 29 images, in descending severity of algorithmic misclassification as diabetic retinopathy. All but one of the 29 images (97%) contained pathology for which clinical and/or lifestyle intervention is indicated. The exception, Image 21, showed an otherwise unremarkable tessellated fundus.

**Fig. 1:**
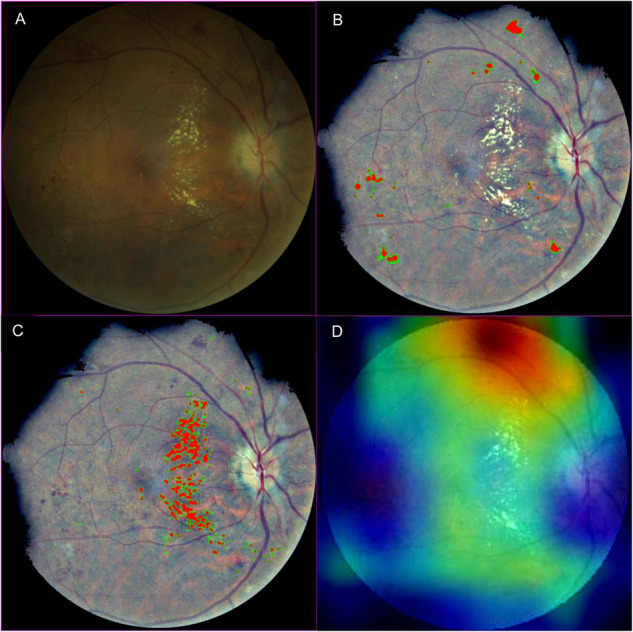
Example image with heatmaps. The original image in Fig. 1 is one of the two images misclassified as referable diabetic retinopathy by all three algorithms (**A**). Of the three heatmaps, two are from Algorithm 2 precisely highlighting blood (**B**) and exudate (**C**), while the heatmap from Algorithm 3 (**D**) did not match the prominent pathology in the image.

Three of the thirteen images flagged by Algorithm 2 had no observable blood or exudate and had no heatmap highlights. The other ten heatmaps precisely highlighted blood (red) and exudate (white), as shown by the example images in Fig. 1. The three heatmaps generated by Algorithm 3 did not coincide with prominent pathology in the images, an example of which is shown in Fig. 1.

### Algorithm-determined referral and clinical utility

**Fig. 2:**
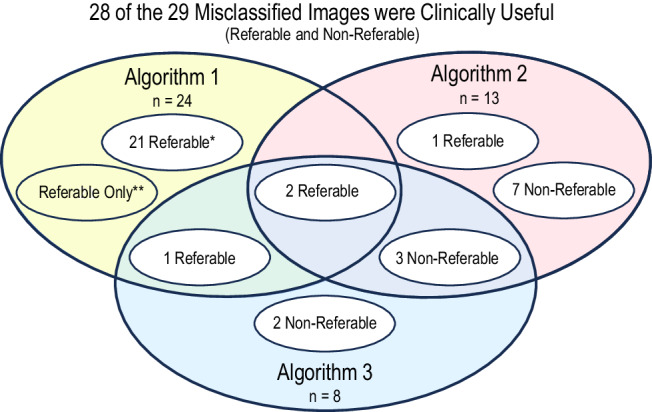
All but 1 of the 29 misclassified images were clinically useful. Figure 2 shows the low inter-algorithm agreement and the distribution of referable and non-referable results within the 29 misclassified images. All but one of the 29 images had clinically useful biomarkers.

Unlike Algorithm 1, Algorithms 2 and 3 provided results for mild *non-referable* diabetic retinopathy. Despite low inter-algorithm agreement demonstrated by the overlap of the 29 false-positive results for diabetic retinopathy in Fig. 2, all but one (97%) of the referable results (misclassified as moderate or severe diabetic retinopathy) and all 12 (100%) of the non-referable results (misclassified as mild diabetic retinopathy) contained clinically significant pathology likely to benefit from intervention.

### Capture counts and number of diseases targeted

**Fig. 3:**
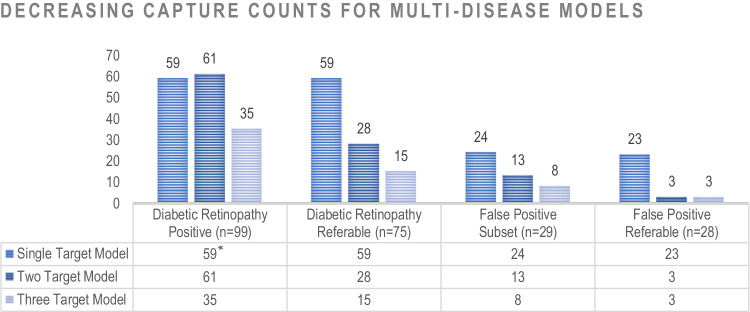
Detection counts for single, double, and triple target algorithms. Figure 3 shows that the algorithms with fewer targets not only output more false positives in the 29 misclassified non-diabetic results and its 28-count referable subset, but also had higher capture counts in the 99 positive diabetic retinopathy results and its 75-count referable subset. It is unlikely that there is an exception in the 99 positive diabetic retinopathy results where the single-target model did not return the most positive results, as the model was not designed to output non-referable results, which were included for the other two models.

The consistent trend of decreasing detection fractions with increasing number of algorithm targets, previously noted for the individual features listed in Table 1, is also evident in the datasets as shown in Fig. 3. Not only did the models with fewer targets output more false positives in the 29 misclassified non-diabetics and its 28-count referable subset, but they also had higher capture counts in the 99 positive diabetic retinopathy results and its 75 count subset. It is unlikely that there is an exception to the overall trend in Fig. 3 where the single-target algorithm flagged fewer images than the two-target algorithm, as the single-target algorithm was not designed to output non-referable results that were included in the total counts of the other two algorithms.

### Hypertensive retinopathy severity and misclassified diabetic retinopathy

**Fig. 4:**
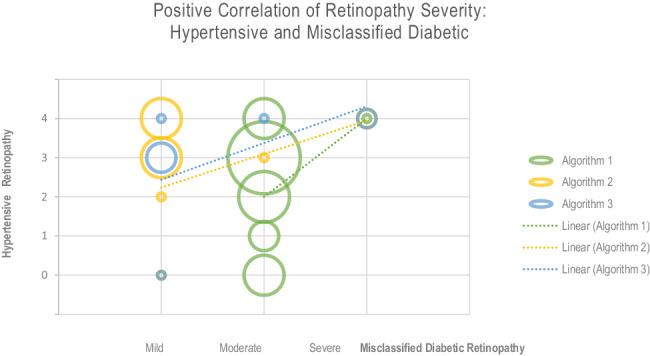
Positive correlation of retinopathy severity: hypertensive and misclassified diabetic. Figure 4 plots the severity of hypertensive retinopathy, according to the Keith-Wagener-Barker scale, increasing from 0 to 4, for the misclassified diabetic retinopathy results of each algorithm: 24 points for Algorithm 1, 13 points for Algorithm 2, and 8 points for Algorithm 3. The count at each intersection is represented by circle sizes and least squares regression lines show the positive correlation for all three algorithms.

Human-assessed hypertensive retinopathy features, artificially classified by the Keith-Wagener-Barker hypertensive retinopathy severity scale [72] increasing from 0 to 4, were plotted on the y-axis as a function of algorithmic diabetic retinopathy misclassification on the x-axis, to create Fig. 4. For each algorithm, Fig. 4 shows how both the frequency and severity of false-positive diabetic retinopathy results in our sample correlate with increasing hypertensive retinopathy features found in the images. Circle size represents the count at each intersection, with least squares regression lines showing the positive correlation for each algorithm.

## Discussion

The study demonstrated that existing deep learning models capture clinically important incidental pathology in fundus photographs misclassified as diabetic retinopathy. As noted, these findings specifically relate to a subset of false-positive results for diabetic retinopathy in an established hypertensive cohort. All three algorithms captured high rates (97%, 100 and 100%) of clinically useful non-target disease, including all (100%) of the results classified as non-referable by the algorithms.

The trend for algorithms targeting fewer diseases to capture more incidental pathology is consistent with the hypothesis that deep learning algorithms with less differential training may have data signals with broader anomaly detection potential. This boosts confidence in suggesting a pivot from the flawed disease-specific comprehensive screening trajectory to generalised anomaly detection with self-supervised deep learning.

Whether the correlation between the severity of hypertensive retinopathy present in the image and the number and severity of misclassifications as diabetic retinopathy for each algorithm arises from biomarker ambiguity, training image comorbidities, and/or untrained broader detection signals embedded in the deep learning pathway remains unknown as the process is hidden. However, clinically, both retinopathies are significant, and comorbidity is common, so all positive results (referable, non-referable and false) have potential immediate utility.

### Similarity of incidental findings in human diabetic screening

Apart from a hypertensive skew, the features and capture rates shown in Table 1 are comparable to the incidental pathologies found in human expert diabetic screening programs [45, 48–55, 73, 74]. In human diabetic retinal screening, hypertensive retinopathy is the most common incidental finding (14 to 34% [45, 48]), followed by drusen (14 to 21% [45]), macular degeneration (0.5 to 18% [45, 48–50]), and retinal vein occlusion (0.7% to 2.2% [45]). Other less common incidental findings include myopic choroidopathy, disc pallor, glaucoma, retinal emboli, geographic atrophy, epiretinal membranes, choroidal nevi, cataract, and posterior capsular opacities [45, 48, 50–55]. Referable incidental pathology in human diabetic screening varies from 24 to 45% [45, 53, 54], often with higher capture rates than the targeted diabetic retinopathy [51, 52]. The similarity of these incidental findings suggests that deep learning false-positive results from diabetic populations may contain a high proportion of the clinically valuable incidental pathology found by human assessment.

### Similarity of false-positive results in general populations

The few published deep learning false-positive results for diabetic retinopathy are based on general population verification datasets and list drusen, exudate, microaneurysm, macular degeneration, venous occlusion, myopic maculopathy, arteriovenous crossing changes, and “normal” [46, 47, 56–59]. These features are a subset of those found in the hypertensive sample and do not necessarily represent the full set of anomalies that may occur for two reasons. First, the “normal” false-positive results may reflect deep learning anomaly detection beyond human observation, which would not be a false-positive error, but rather a subclinical detection and verification failure. Second, the selected anomalies are presented as plausible explanations for misclassification errors rather than a representation of the full set of features present in the false-positive images. Despite limited data, two algorithms reported referable pathology rates for their false-positive results of 80% [59] and 92% [47], suggesting that further investigation of false-positive results in wider populations may prove clinically useful.

### Differential diagnosis of hypertensive and diabetic retinopathies

Although algorithmic pathways are hidden, the sensitivity of diabetic algorithms to hypertensive retinopathy may arise from the artificial and incomplete human classification of training images due to ambiguous biomarkers between the retinopathies [75]. This ambiguity appears to increase for earlier shared biomarkers, such as capillary rarefaction and reduced vessel density as seen with OCTA [76, 77] and is demonstrated algorithmically by a 6% specificity rise for Algorithm 2 when mild diabetic retinopathy detection was excluded [68, 69]. The correlation found between the degree of hypertensive retinopathy and the severity of diabetic misclassification not only shows that deep learning models can capture useful non-target pathology as false-positive results, but also raises the possibility that human limitations in biomarker knowledge may lead to algorithmic misclassification, inhibiting target-specific algorithm development.

### Expanding deep learning utility

Algorithm target-specificity hinders progress towards autonomous comprehensive screening not only from misclassification and missed incidental disease, but also from inter-algorithm inconsistency and ethical bias. Disease-specific deep learning models are trained to make innumerable comparisons to define volume-derived baseline data to which inputs may be matched. Inconsistency and ethical concerns arise from the variable and biased human selection of training data and supervision used to define the hidden baseline data. Although saliency analysis, a technique of progressively sectioning the fundus to isolate anomalies, has been successful in refining heatmap outputs to theorise biomarkers used by an algorithm [78], and generative artificial intelligence modification of hypothesised features can further isolate potential data signals used to determine algorithm output [79], the baseline data is not exposed and outputs still vary between algorithms. This poses regulatory challenges, such as transparency of embedded normative data as applied to OCT [80–82] and raises ethical issues of input bias.

The consistency of the distribution of incidental findings found in false positive results in this study and in screening diabetic and general populations with other deep learning models increases confidence in the hypothesis that deep learning models have data signals capable of broader anomaly detection. As deep learning models are based on pattern recognition and discrimination, training on the existing vast and diverse repository of healthy retinal images to define normative data may generate a more comprehensive, consistent, and generalisable model. To differentiate pathology from anomalies arising from lighting, positioning, media opacities, and artefacts [83] without artificial classification and human supervisory bias, an autoencoding technique, known as *self-supervision*, has demonstrated success in distinguishing not only artefacts and media opacities, but also tessellation [84]. A variety of self-supervised feature learning models have been developed for medical imaging [85] including one using OCT images that is capable of general anomaly detection without differential diagnostic output [86]. Such initial triage could be of immediate benefit for those with access to OCT, but exceptional access would be realised when sufficient datasets and biomarker knowledge are available to use external eye images [87, 88]. Until then, self-supervised learning models to analyse fundus photographs could not only address scope, consistency, and ethical issues, but also provide opportunities for novel associations and biomarker discovery. A foundational model has now become publicly available, offering anomaly detection without diagnostic classification, ready for differential diagnosis development and labelling [89].

### Strengths and limitations

To strengthen internal validity, exclusively non-diabetic participants comprised the subset of false-positive results for diabetic retinopathy. This not only eliminated potential human grading errors, but also minimised false-positive misclassification of true positive images arising from algorithmic subclinical detection of diabetic vascular changes [69], such as capillary damage [76, 77] which are known to exist prior to threshold glycated haemoglobin indicators in both prediabetics [90] and diabetics [91].

However, these results may have limited external generalisability as they represent a single site with potential selection biases related to hypertensive status, diabetic status, demographics, and voluntary participation. The extent of verified retinal pathology in the wider clinical population is unknown, and the high capture rate of clinically significant pathology observed in this at-risk subset of false-positive results may not be broadly representative.

## Conclusion

The deep learning models in this study captured high rates of clinically significant incidental pathology in the misclassified non-diabetic results studied, raising the possibility of immediate clinical use of false positives in broader (beyond diabetic) screening and in other (beyond hypertensive) populations.

In the quest for full-scope autonomous screening, current development combining disease-specific models is flawed by limitations of human biomarker knowledge and the inability to train for rare and novel diseases. Conceivably, incidental capture may approach full-scope disease detection, but the study found that more incidental disease was captured by less trained models, which better aligns with using self-supervised deep learning to expand biomarker knowledge and as an alternate route to achieve comprehensive autonomous retinal screening.

## Summary

### What was known before

- Deep learning models can detect target retinal diseases earlier and more accurately than human experts.
- Deep learning models are not trained to detect important incidental pathology found by human screening.

### What this study adds

- Targeted deep learning retinal analysis may capture high rates of clinically useful non-target pathology as false-positive results.
- Development of self-supervised deep learning models is proposed as an alternate pathway to achieve comprehensive autonomous retinal screening.

## Supplementary information

Supplementary Table 1

Supplementary Table 1 legend

## Data Availability

The data supporting the findings of this study are not publicly available due to participant privacy protections but are available from the corresponding author upon reasonable request.

## References

[1] Abràmoff MD, Lavin PT, Birch M, Shah N, Folk JC. Pivotal trial of an autonomous AI-based diagnostic system for detection of diabetic retinopathy in primary care offices. NPJ Digit Med. 2018;1:1–8. 31304320 10.1038/s41746-018-0040-6 PMC6550188

[2] Keel S, Li Z, Scheetz J, Robman L, Phung J, Makeyeva G, et al. Development and validation of a deep-learning algorithm for the detection of neovascular age-related macular degeneration from colour fundus photographs. Clin Exp Ophthalmol. 2019;47:1009–18. 31215760 10.1111/ceo.13575

[3] Liu H, Li L, Wormstone IM, Qiao C, Zhang C, Liu P, et al. Development and validation of a deep learning system to detect glaucomatous optic neuropathy using fundus photographs. JAMA Ophthalmol. 2019;137:1353–60. 31513266 10.1001/jamaophthalmol.2019.3501 PMC6743057

[4] Wang R, He J, Chen Q, Ye L, Sun D, Yin L, et al. Efficacy of a Deep Learning System for Screening Myopic Maculopathy Based on Color Fundus Photographs. Ophthalmol Ther. 2023;12:469–84. 36495394 10.1007/s40123-022-00621-9 PMC9735275

[5] Vaghefi E, Squirrell D, Yang S, An S, Xie L, Durbin MK, et al. Development and validation of a deep-learning model to predict 10-year ASCVD risk from retinal images using the UK Biobank and EyePACS 10K datasets. Cardiovasc Digit Health J. 2024;5:59-69. 38765618 10.1016/j.cvdhj.2023.12.004 PMC11096659

[6] Liu X, Ali TK, Singh P, Shah A, McKinney SM, Ruamviboonsuk P, et al. Deep learning to detect OCT-derived diabetic macular edema from color retinal photographs: a multicenter validation study. Ophthalmol Retin. 2022;6:398–410. 10.1016/j.oret.2021.12.021 34999015

[7] Krause J, Gulshan V, Rahimy E, Karth P, Widner K, Corrado GS, et al. Grader variability and the importance of reference standards for evaluating machine learning models for diabetic retinopathy. Ophthalmology. 2018;125:1264–72. 29548646 10.1016/j.ophtha.2018.01.034

[8] Wu Z, Pfau M, Blodi BA, Holz FG, Jaffe GJ, Liakopoulos S, et al. OCT Signs of early atrophy in age-related macular degeneration: Interreader agreement: Classification of atrophy meetings report 6. Ophthalmol Retin. 2022;6:4–14. 10.1016/j.oret.2021.03.008 33766801

[9] Abdellaoui T, Malek Y, Brarou H, Elasri F, Mouzari Y, Reda K, et al. Quantitative assessment of optic disc photographs in normal and open-angle glaucoma patients. Ophthalmol J. 2022;7:12–9.

[10] Heydon P, Egan C, Bolter L, Chambers R, Anderson J, Aldington S, et al. Prospective evaluation of an artificial intelligence-enabled algorithm for automated diabetic retinopathy screening of 30000 patients. Br J Ophthalmol. 2021;105:723–8. 32606081 10.1136/bjophthalmol-2020-316594 PMC8077216

[11] Foot B, MacEwen C. Surveillance of sight loss due to delay in ophthalmic treatment or review: frequency, cause and outcome. Eye. 2017;31:771–5. 28128796 10.1038/eye.2017.1 PMC5437335

[12] Resnikoff S, Lansingh VC, Washburn L, Felch W, Gauthier T-M, Taylor HR, et al. Estimated number of ophthalmologists worldwide (International Council of Ophthalmology update): will we meet the needs? Br J Ophthalmol. 2020;104:588–92. 31266774 10.1136/bjophthalmol-2019-314336 PMC7147181

[13] V SA, Sivaswamy J, editors. Matching the characteristics of fundus and smartphone camera images. *16th Int Symp Biomed Imaging (ISBI 2019)*; 2019; Venice, Italy: IEEE.

[14] Lim G, Cheng Y, Hsu W, Lee ML, editors. Integrated optic disc and cup segmentation with deep learning. *2015 IEEE 27th Int Conf Tools Art Intel (ICTAI)*; 2015: IEEE.

[15] Elloumi Y, Mbarek MB, Boukadida R, Akil M, Bedoui MH, editors. Fast and accurate mobile-aided screening system of moderate diabetic retinopathy. *13th Intl Conf Mach Vis*; 2021: SPIE.

[16] Gulshan V, Peng L, Coram M, Stumpe MC, Wu D, Narayanaswamy A, et al. Development and validation of a deep learning algorithm for detection of diabetic retinopathy in retinal fundus photographs. JAMA. 2016;316:2402–10. 27898976 10.1001/jama.2016.17216

[17] Olvera-Barrios A, Heeren TF, Balaskas K, Chambers R, Bolter L, Egan C, et al. Diagnostic accuracy of diabetic retinopathy grading by an artificial intelligence-enabled algorithm compared with a human standard for wide-field true-colour confocal scanning and standard digital retinal images. Br J Ophthalmol. 2021;105:265–70. 32376611 10.1136/bjophthalmol-2019-315394

[18] Tufail A, Rudisill C, Egan C, Kapetanakis VV, Salas-Vega S, Owen CG, et al. Automated diabetic retinopathy image assessment software: diagnostic accuracy and cost-effectiveness compared with human graders. Ophthalmology. 2017;124:343–51. 28024825 10.1016/j.ophtha.2016.11.014

[19] Mokhashi N, Grachevskaya J, Cheng L, Yu D, Lu X, Zhang Y, et al. A comparison of artificial intelligence and human diabetic retinal image interpretation in an urban health system. J Diabetes Sci Technol. 2021;16:1003–7. 33719599 10.1177/1932296821999370 PMC9264425

[20] Liu C, Wang W, Li Z, Jiang Y, Han X, Ha J, et al., editors. Biological age estimated from retinal imaging: a novel biomarker of aging. *Int Conf Med Imag Comp Assist Intervent*; 2019: Springer.

[21] Varadarajan AV, Poplin R, Blumer K, Angermueller C, Ledsam J, Chopra R, et al. Deep learning for predicting refractive error from retinal fundus images. Invest Ophthalmol Vis Sci. 2018;59:2861–8. 30025129 10.1167/iovs.18-23887

[22] Poplin R, Varadarajan AV, Blumer K, Liu Y, McConnell MV, Corrado GS, et al. Prediction of cardiovascular risk factors from retinal fundus photographs via deep learning. Nat Biomed Eng. 2018;2:158–64. 31015713 10.1038/s41551-018-0195-0

[23] Rim TH, Lee G, Kim Y, Tham YC, Lee CJ, Baik SJ, et al. Prediction of systemic biomarkers from retinal photographs: development and validation of deep-learning algorithms. Lancet Digit Health. 2020;2:e526–36. 33328047 10.1016/S2589-7500(20)30216-8

[24] Cheung CY, Xu D, Cheng C-Y, Sabanayagam C, Tham Y-C, Yu M, et al. A deep-learning system for the assessment of cardiovascular disease risk via the measurement of retinal-vessel calibre. Nat Biomed Eng. 2021;5:498–508. 33046867 10.1038/s41551-020-00626-4

[25] Mitani A, Huang A, Venugopalan S, Corrado GS, Peng L, Webster DR, et al. Detection of anaemia from retinal fundus images via deep learning. Nat Biomed Eng. 2020;4:18–27. 31873211 10.1038/s41551-019-0487-z

[26] Appaji A, Harish V, Korann V, Devi P, Jacob A, Padmanabha A, et al. Deep learning model using retinal vascular images for classifying schizophrenia. Schizophr Res. 2022;241:238–43. 35176722 10.1016/j.schres.2022.01.058

[27] Hu W, Wang W, Wang Y, Chen Y, Shang X, Liao H, et al. Retinal age gap as a predictive biomarker of future risk of Parkinson’s disease. Age Ageing. 2022;51:62. 10.1093/ageing/afac062 PMC8966015 35352798

[28] Tian J, Smith G, Guo H, Liu B, Pan Z, Wang Z, et al. Modular machine learning for Alzheimer’s disease classification from retinal vasculature. Sci Rep. 2021;11:1–11. 33420208 10.1038/s41598-020-80312-2 PMC7794289

[29] Chang J, Ko A, Park SM, Choi S, Kim K, Kim SM, et al. Association of cardiovascular mortality and deep learning-funduscopic atherosclerosis score derived from retinal fundus images. Am J Ophthalmol. 2020;217:121–30. 32222370 10.1016/j.ajo.2020.03.027

[30] Son J, Shin JY, Chun EJ, Jung K-H, Park KH, Park SJ. Predicting high coronary artery calcium score from retinal fundus images with deep learning algorithms. Transl Vis Sci Technol. 2020;9:28. 33184590 10.1167/tvst.9.2.28 PMC7410115

[31] Zhu Z, Chen Y, Wang W, Wang Y, Hu W, Shang X, et al. Association of retinal age gap with arterial stiffness and incident cardiovascular disease. Stroke. 2022;53:3320–8. 35880520 10.1161/STROKEAHA.122.038809

[32] Lim G, Lim ZW, Xu D, Ting DS, Wong TY, Lee ML, et al. editors. Feature isolation for hypothesis testing in retinal imaging: an ischemic stroke prediction case study. AAAI Conf Art Intel. 2019.

[33] Li B, Chen H, Zhang B, Yuan M, Jin X, Lei B, et al. Development and evaluation of a deep learning model for the detection of multiple fundus diseases based on colour fundus photography. Br J Ophthalmol. 2022;106:1079–86. 33785508 10.1136/bjophthalmol-2020-316290

[34] Cen L-P, Ji J, Lin J-W, Ju S-T, Lin H-J, Li T-P, et al. Automatic detection of 39 fundus diseases and conditions in retinal photographs using deep neural networks. Nat Comm. 2021;12:1–13. 10.1038/s41467-021-25138-w PMC8355164 34376678

[35] Son J, Shin JY, Kim HD, Jung K-H, Park KH, Park SJ. Development and validation of deep learning models for screening multiple abnormal findings in retinal fundus images. Ophthalmology. 2020;127:85–94. 31281057 10.1016/j.ophtha.2019.05.029

[36] Kang EY-C, Yeung L, Lee Y-L, Wu C-H, Peng S-Y, Chen Y-P, et al. A multimodal imaging–based deep learning model for detecting treatment-requiring retinal vascular diseases: model development and validation study. JMIR Med Info. 2021;9:e28868. 10.2196/28868 PMC8204240 34057419

[37] Hutchinson A, McIntosh A, Peters J, O’keeffe C, Khunti K, Baker R, et al. Effectiveness of screening and monitoring tests for diabetic retinopathy–a systematic review. Diabet Med. 2000;17:495–506. 10972578 10.1046/j.1464-5491.2000.00250.x

[38] Long AN, Dagogo‐Jack S. Comorbidities of diabetes and hypertension: mechanisms and approach to target organ protection. J Clin Hypertens. 2011;13:244–51. 10.1111/j.1751-7176.2011.00434.x PMC3746062 21466619

[39] Wong TY, McIntosh R. Hypertensive retinopathy signs as risk indicators of cardiovascular morbidity and mortality. Br Med Bullet. 2005;73:57–70. 10.1093/bmb/ldh050 16148191

[40] Ding J, Wai KL, McGeechan K, Kawasaki R, Xie J, Klein R, et al. Retinal vascular caliber and the development of hypertension: a meta-analysis of individual participant data. J Hyperten. 2014;32:207. 10.1097/HJH.0b013e32836586f4 PMC4120649 24322199

[41] Unger T, Borghi C, Charchar F, Khan NA, Poulter NR, Prabhakaran D, et al. 2020 International Society of Hypertension global hypertension practice guidelines. Hypertension. 2020;75:1334–57. 32370572 10.1161/HYPERTENSIONAHA.120.15026

[42] Nguyen TN, Chow CK. Global and national high blood pressure burden and control. Lancet. 2021;398:932–3. 34450082 10.1016/S0140-6736(21)01688-3

[43] World Health Organization. Hypertension: WHO; 2021 [updated 25 Aug 2021]. Available from: https://www.who.int/news-room/fact-sheets/detail/hypertension.

[44] Gabb GM, Mangoni AA, Anderson CS, Cowley D, Dowden JS, Golledge J, et al. Guideline for the diagnosis and management of hypertension in adults—2016. Med J Aust. 2016;205:85–9. 27456450 10.5694/mja16.00526

[45] Ramachandran N, Schmiedel O, Vaghefi E, Hill S, Wilson G, Squirrell D. Evaluation of the prevalence of non-diabetic eye disease detected at first screen from a single region diabetic retinopathy screening program: a cross-sectional cohort study in Auckland, New Zealand. BMJ open. 2021;11:e054225. 34907067 10.1136/bmjopen-2021-054225 PMC8672006

[46] Kanagasingam Y, Xiao D, Vignarajan J, Preetham A, Tay-Kearney M-L, Mehrotra A. Evaluation of artificial intelligence–based grading of diabetic retinopathy in primary care. JAMA Netw Open. 2018;1:e182665. 30646178 10.1001/jamanetworkopen.2018.2665 PMC6324474

[47] Li Z, Keel S, Liu C, He Y, Meng W, Scheetz J, et al. An automated grading system for detection of vision-threatening referable diabetic retinopathy on the basis of color fundus photographs. Diabet Care. 2018;41:2509–16. 10.2337/dc18-0147 30275284

[48] Mastropasqua L, Perilli R, D’Aloisio R, Toto L, Mastropasqua A, Donato S, et al. Why miss the chance? Incidental findings while telescreening for diabetic retinopathy. Ophthalmic Epidemiol. 2020;27:237–45. 31958252 10.1080/09286586.2020.1715450

[49] Gangwani R, Lai WW, Sum R, McGhee SM, Chan CW, Hedley AJ, et al. The incidental findings of age-related macular degeneration during diabetic retinopathy screening. Graefes Arch Clin Exp Ophthalmol. 2014;252:723–9. 24281784 10.1007/s00417-013-2530-1

[50] Cotter K, Holbrook K, Yates PA. Incidental findings identified through diabetic retinopathy screening and potential impact on computer automated diabetic retinopathy reading algorithms. Invest Ophthalmol Vis Sci. 2014;55:4824.

[51] Nielsen N, Jackson C, Spurling G, Cranstoun P. Nondiabetic retinal pathology: prevalence in diabetic retinopathy screening. Aust Fam Physic. 2011;40:529–32. 21743863

[52] Hadziahmetovic M, Amason J, Lee T, Cousins S. Remote diagnosis of referable retinal pathology in diabetic patients visiting primary care clinic. Invest Ophthalmol Vis Sci. 2021;62:1147.

[53] Lee T, Amason J, Del Risco A, Kim J-B, Cousins SW, Hadziahmetovic M. Incidence of referable retinal disease in diabetic patients at a primary care practice. J Vitreoret Dis. 2022;6:138–46. 10.1177/24741264211044223 PMC9976004 37008662

[54] Boucher MC, Desroches G, Garcia-Salinas R, Kherani A, Maberley D, Olivier S, et al. Teleophthalmology screening for diabetic retinopathy through mobile imaging units within Canada. Canad J Ophthalmol. 2008;43:658–68. 19020631 10.3129/i08-120

[55] Owsley C, McGwin G, Lee DJ, Lam BL, Friedman DS, Gower EW, et al. Diabetes eye screening in urban settings serving minority populations: detection of diabetic retinopathy and other ocular findings using telemedicine. JAMA Ophthalmol. 2015;133:174–81. 25393129 10.1001/jamaophthalmol.2014.4652 PMC4479273

[56] Wongchaisuwat N, Trinavarat A, Rodanant N, Thoongsuwan S, Phasukkijwatana N, Prakhunhungsit S, et al. In-person verification of deep learning algorithm for diabetic retinopathy screening using different techniques across fundus image devices. Transl Vis Sci Technol. 2021;10:17. 34767624 10.1167/tvst.10.13.17 PMC8590162

[57] Lu L, Ren P, Lu Q, Zhou E, Yu W, Huang J, et al. Analyzing fundus images to detect diabetic retinopathy (DR) using deep learning system in the Yangtze River delta region of China. Ann Transl Med. 2021;9:226. 33708853 10.21037/atm-20-3275 PMC7940941

[58] Ruamviboonsuk P, Tiwari R, Sayres R, Nganthavee V, Hemarat K, Kongprayoon A, et al. Real-time diabetic retinopathy screening by deep learning in a multisite national screening programme: a prospective interventional cohort study. Lancet Digit Health. 2022;4:235–44. 10.1016/S2589-7500(22)00017-6 35272972

[59] Li N, Ma M, Lai M, Gu L, Kang M, Wang Z, et al. A stratified analysis of a deep learning algorithm in the diagnosis of diabetic retinopathy in a real‐world study. J Diabet. 2022;14:111–20. 10.1111/1753-0407.13241 PMC9060020 34889059

[60] Guo S, Yin S, Tse G, Li G, Su L, Liu T. Association between caliber of retinal vessels and cardiovascular disease: a systematic review and meta-analysis. Curr Atheroscler Rep. 2020;22:1–13. 10.1007/s11883-020-0834-2 32440852

[61] Arsalan M, Haider A, Lee YW, Park KR. Detecting retinal vasculature as a key biomarker for deep learning-based intelligent screening and analysis of diabetic and hypertensive retinopathy. Exp Syst Appl. 2022;200:117009.

[62] Hua D, Xu Y, Zeng X, Yang N, Jiang M, Zhang X, et al. Use of optical coherence tomography angiography for assessment of microvascular changes in the macula and optic nerve head in hypertensive patients without hypertensive retinopathy. Microvasc Res. 2020;129:103969. 31874131 10.1016/j.mvr.2019.103969

[63] Arsalan M, Haider A, Choi J, Park KR. Diabetic and hypertensive retinopathy screening in fundus images using artificially intelligent shallow architectures. J Pers Med 2022;12:7. 10.3390/jpm12010007 PMC8777982 35055322

[64] Egan BM, Zhao Y, Brzezinski WA Epidemiology of hypertension in diabetes. *Diabet Hyperten*: Springer; 2012. p. 1-14.

[65] Frost S, Nolde JM, Chan J, Joyson A, Gregory C, Carnagarin R, et al. Retinal capillary rarefaction is associated with arterial and kidney damage in hypertension. Sci Rep. 2021;11:1–10. 33441624 10.1038/s41598-020-79594-3 PMC7806760

[66] Nolde JM, Frost S, Kannenkeril D, Lugo-Gavidia LM, Chan J, Joyson A, et al. Capillary vascular density in the retina of hypertensive patients is associated with a non-dipping pattern independent of mean ambulatory blood pressure. J Hyperten. 2021;39:1826–34. 10.1097/HJH.0000000000002863 34397628

[67] International Council of Ophthalmology. *ICO Guidelines for Diabetic Eyecare: ICO*; 2017 [updated Jan 2017]. Available from: https://icoph.org/eye-care-delivery/ico-international-clinical-guidelines-and-resources/.

[68] Sánchez-Gutiérrez V, Hernández-Martínez P, Muñoz-Negrete FJ, Engelberts J, Luger AM, van Grinsven MJ Performance of a deep learning system for detection of referable diabetic retinopathy in real clinical settings. arXiv preprint arXiv:220505554. 2022.

[69] González‐Gonzalo C, Sánchez‐Gutiérrez V, Hernández‐Martínez P, Contreras I, Lechanteur YT, Domanian A, et al. Evaluation of a deep learning system for the joint automated detection of diabetic retinopathy and age‐related macular degeneration. Acta Ophthalmol. 2020;98:368–77. 31773912 10.1111/aos.14306 PMC7318689

[70] Li Z, He Y, Keel S, Meng W, Chang RT, He M. Efficacy of a deep learning system for detecting glaucomatous optic neuropathy based on color fundus photographs. Ophthalmology. 2018;125:1199–206. 29506863 10.1016/j.ophtha.2018.01.023

[71] Royal Australian and New Zealand College of Ophthalmologists. *RANZCO Screening and Referral Pathway for Diabetic Retinopathy: RANZCO*; 2016 [updated 2018]. Available from: https://ranzco.edu/wp-content/uploads/2018/11/RANZCO-Referral-pathway-for-DR-2016.pdf.

[72] Keith NM. Some different types of essential hypertension: their course and prognosis. Am J Med Sci. 1939;197:332–43. 10.1097/00000441-197412000-00004 4616627

[73] Gangwani RA, McGhee SM, Lai JS, Chan CK, Wong D. Detection of glaucoma and its association with diabetic retinopathy in a diabetic retinopathy screening program. J Glaucoma. 2016;25:101–5. 25264989 10.1097/IJG.0000000000000138

[74] Treacy MP, O’Neill EC, Murphy M, O’Toole L, Delaney Y, O’Brien C, et al. Opportunistic detection of glaucomatous optic discs within a diabetic retinopathy screening service. Eur J Ophthalmol. 2016;26:315–20. 26692063 10.5301/ejo.5000722

[75] Arsalan M, Haider A, Choi J, Park KR. Diabetic and hypertensive retinopathy screening in fundus images using artificially intelligent shallow architectures. J Pers Med. 2021;12:7. 35055322 10.3390/jpm12010007 PMC8777982

[76] Alibhai AY, Moult EM, Shahzad R, Rebhun CB, Moreira-Neto C, McGowan M, et al. Quantifying microvascular changes using OCT angiography in diabetic eyes without clinical evidence of retinopathy. Ophthalmol Retin. 2018;2:418–27. 10.1016/j.oret.2017.09.011 PMC6391050 30820483

[77] Takase N, Nozaki M, Kato A, Ozeki H, Yoshida M, Ogura Y. Enlargement of foveal avascular zone in diabetic eyes evaluated by en face optical coherence tomography angiography. Retina. 2015;35:2377–83. 26457396 10.1097/IAE.0000000000000849

[78] Mueller S, Wintergerst MWM, Falahat P, Holz FG, Schaefer C, Schahab N, et al. Multiple instance learning detects peripheral arterial disease from high-resolution color fundus photography. Sci Rep. 2022;12:1389. 35082343 10.1038/s41598-022-05169-z PMC8792038

[79] Lang O, Yaya-Stupp D, Traynis I, Cole-Lewis H, Bennett CR, Lyles C, et al. Using generative AI to investigate medical imagery models and datasets. eBioMedicine. 2024;102:105075. 38565004 10.1016/j.ebiom.2024.105075 PMC10993140

[80] Optovue Inc. *RTVue with normative database: FDA*; 2010 [updated 15 Sep 2010]. Available from: https://www.accessdata.fda.gov/cdrh_docs/pdf10/K101505.pdf.

[81] Carl Zeiss Meditec. *Cirrus HD-OCT with retinal nerve fiber layer and macular normative databases: FDA*; 2009 [updated 5 May 2009]. Available from: https://www.accessdata.fda.gov/cdrh_docs/pdf8/K083291.pdf.

[82] Heidelberg Engineering. *Spectralis HRA plus OCT and variants: FDA*; 2016 [updated 6 May 2016]. Available from: https://www.accessdata.fda.gov/cdrh_docs/pdf15/K152205.pdf.

[83] Wolff G, Sayres R, Gulshan V, Widner K, Krause J, Jadeja D, et al. Challenges in evaluating clinical deployments of deep learning assisted diagnostics for diabetic retinopathy screening. Invest Ophthalmol Vis Sci. 2020;61:2045.

[84] Yellapragada B, Hornauer S, Snyder K, Yu S, Yiu G. Self-supervised feature learning and phenotyping for assessing age-related macular degeneration using retinal fundus images. Ophthalmol Retin. 2022;6:116–29. 10.1016/j.oret.2021.06.010 PMC9482819 34217854

[85] Huang S-C, Pareek A, Jensen M, Lungren MP, Yeung S, Chaudhari AS. Self-supervised learning for medical image classification: a systematic review and implementation guidelines. NPJ Digit Med. 2023;6:74. 37100953 10.1038/s41746-023-00811-0 PMC10131505

[86] Li X, Jia M, Islam MT, Yu L, Xing L. Self-supervised feature learning via exploiting multi-modal data for retinal disease diagnosis. IEEE Trans Med Imaging. 2020;39:4023–33. 32746140 10.1109/TMI.2020.3008871

[87] Babenko B, Mitani A, Traynis I, Kitade N, Singh P, Maa AY, et al. Detection of signs of disease in external photographs of the eyes via deep learning. Nat Biomed Eng. 2022:6:1–14. 35352000 10.1038/s41551-022-00867-5 PMC8963675

[88] Babenko B, Traynis I, Chen C, Singh P, Uddin A, Cuadros J, et al. A deep learning model for novel systemic biomarkers in photographs of the external eye: a retrospective study. Lancet Digit Health. 2023;5:E257–64. 36966118 10.1016/S2589-7500(23)00022-5 PMC11818944

[89] Zhou Y, Chia MA, Wagner SK, Ayhan MS, Williamson DJ, Struyven RR, et al. A foundation model for generalizable disease detection from retinal images. Nature. 2023;622:156–63. 37704728 10.1038/s41586-023-06555-x PMC10550819

[90] Wong TY, Barr EL, Tapp RJ, Harper CA, Taylor HR, Zimmet PZ, et al. Retinopathy in persons with impaired glucose metabolism: the Australian Diabetes Obesity and Lifestyle (AusDiab) study. Am J Ophthalmol. 2005;140:1157–9. 16376677 10.1016/j.ajo.2005.07.030

[91] Kannenkeril D, Nolde JM, Kiuchi MG, Carnagarin R, Lugo-Gavidia LM, Chan J, et al. Retinal capillary damage is already evident in patients with hypertension and prediabetes and associated with HbA1c levels in the nondiabetic range. Diabet Care. 2022;45:1472–5. 10.2337/dc21-1569 35344581

