# Supplementary material for: Ocular biomarkers: useful incidental findings by deep learning algorithms in fundus photographs
Source: Eye (Lond). 2024 May 11;38(13):2581–8. doi: 10.1038/s41433-024-03085-2 (PMC11385472; doi:10.1038/s41433-024-03085-2)
Supplement: Supplementary file 1 — Supplementary Table 1 [file 41433_2024_3085_MOESM1_ESM.pdf]

Features of the 29 Images Misclassified as Diabetic Retinopathy: Ordered by Descending Severity of Misclassification

Image	Features	Model 1	Model 2	Model 3
1	Hypertensive vascular remodelling, haemorrhage, macular lipid exudate, disc swelling	Severe	Severe	Severe
2	Retinal vein occlusion with macular lipid exudate	Moderate	Severe	Severe
3	Retinal vein occlusion with macular lipid exudate	Moderate	Mild	Moderate
4	Retinal vein occlusion with macular lipid exudate	Moderate	Mild	Mild
5	Widened fovea with unusual macular appearance with lipid exudate	Moderate	Mild	Mild
6	Mild hypertensive retinopathy with branch venous occlusion and macular degeneration	Moderate		Mild
7	Venous occlusion with macular lipid exudate	Moderate	Mild	
8	Hypertensive vascular remodelling with haemorrhage		Moderate	Mild
9	Hypertensive vascular remodelling	Moderate	Mild	
10	Hypertensive vascular remodelling and retinal pigment epithelial changes of the macula	Moderate	Mild	
11	Hypertensive vascular remodelling with ischemia		Mild	Mild
12	Hypertensive vascular remodelling with ischemia and macular microaneurysm	Moderate		
13	Hypertensive vascular remodelling and suspect myopic choroidopathy	Moderate		
14	Hypertensive vascular remodelling and myopic maculopathy	Moderate		
15	Hypertensive vascular remodelling with ischemia	Moderate		
16	Hypertensive vascular remodelling	Moderate		
17	Macular degeneration	Moderate		
18	Hypertensive vascular remodelling and scattered drusen	Moderate		
19	Macular degeneration with peripheral drusen and choroidal naevus	Moderate		
20	Hypertensive vascular remodelling and scattered drusen	Moderate		
21	(Non-referable) Fundus tessellation: Mild myopic and/or blonde fundus	Moderate		
22	Macular degeneration	Moderate		
23	Suspect choroiditis or Malattia Leventinese	Moderate		
24	Retinal pigment epithelium disruption of the macula/suspect central serous retinopathy	Moderate		
25	Cuticular drusen associated with membranoproliferative glomerulonephritis	Moderate		
26	Retinal pigment epithelium disruption of the macula and cataract	Moderate		
27	Retinal vein occlusion		Mild	
28	Peau d'orange with angioid streaks associated with pseudoxanthoma elasticum		Mild	
29	Hypertensive vascular remodelling with haemorrhage and ischemia		Mild	
