# Supplementary material for: Ocular biomarkers: useful incidental findings by deep learning algorithms in fundus photographs
Source: Eye (Lond). 2024 May 11;38(13):2581–8. doi: 10.1038/s41433-024-03085-2 (PMC11385472; doi:10.1038/s41433-024-03085-2)
Supplement: Supplementary file 2 — Supplementary Table 1 legend [file 41433_2024_3085_MOESM2_ESM.docx]

Supplementary Table 1: Incidental Pathology and Features of the 29 Misclassified Images with Algorithm Results
